# Effects of Phosphorus-Solubilizing *Rhodopseudomonas palustris* VNW64, VNS89, TLS06, and VNW02 on Phosphorus Nutrient Dynamics and Yield of Hybrid Maize Grown in In-Dyke Alluvial Soil

**DOI:** 10.3390/plants15152312

**Published:** 2026-07-28

**Authors:** Nguyen Quoc Khuong, Pham Thi Phuong Thao

**Affiliations:** 1 Faculty of Crop Science, College of Agriculture, Can Tho University, Can Tho 94000, Vietnam; nqkhuong@ctu.edu.vn; 2 Faculty of Physiology-Biochemistry, College of Agriculture, Can Tho University, Can Tho 94000, Vietnam

**Keywords:** hybrid maize, in-dyke alluvial soil, phosphorus-solubilizing bacteria, purple nonsulfur bacteria

## Abstract

Phosphorus (P) fixation in in-dyke alluvial soils can limit maize productivity and reduce P fertilizer-use efficiency. This study evaluated whether P-solubilizing purple nonsulfur bacteria (PNSB), comprising *Rhodopseudomonas palustris* VNW64, VNS89, TLS06, and VNW02, could improve soil P dynamics, P uptake, growth, and yield of hybrid maize. A pot experiment using soil from Chau Phu commune, An Giang province, Vietnam, was arranged in a randomized complete block design with ten treatments and four replicates. Treatments combined recommended N and K fertilization with 100, 75, 50, or 25% of the recommended P rate, with or without PNSB, plus unfertilized controls. PNSB supplementation increased soil pH, NH_4_^+^ concentration, and soluble P content, while reducing insoluble Al-P, Fe-P, and Ca-P fractions. Inoculated treatments also enhanced P uptake, biomass accumulation, growth traits, SPAD index, yield components, and grain yield compared with the corresponding non-inoculated treatments. Applying 75% of the recommended P rate with PNSB produced higher grain yield and P uptake than full P fertilization without PNSB. Thus, *R. palustris* VNW64, VNS89, TLS06, and VNW02 can reduce chemical P fertilizer input by 25% while sustaining or improving hybrid maize performance in in-dyke alluvial soil.

## 1. Introduction

Maize is a globally important cereal crop used for food, feed, and industrial raw materials, and its productivity strongly depends on balanced nutrient management [1]. It provides many natural compounds, such as carotenoids, phenolics, and phytosterols [2,3], which can reduce the risk of chronic diseases [4]. In Vietnam, An Giang has the largest hybrid maize cultivation area in the Mekong Delta, with 6.0 thousand ha [5], concentrated in dyked areas. However, closed dyke systems have affected the annual deposition of alluvium into fields, leading to a reduced capacity to supply essential nutrients to crops and resulting in reduced crop productivity [6]. Therefore, improving nutrient-use efficiency, particularly phosphorus (P)-use efficiency, is important for sustaining maize production in in-dyke alluvial soils.

Phosphorus (P) is essential for many crops and plays a central role in the formation of nucleic acids and ATP, the energy currency of cells [7]. However, P exists in in-dyke alluvial soil in insoluble forms such as Ca-P [8]. Many bacterial species have been selected to improve P-use efficiency [9]. For example, *Enterobacter vonholyi* Y16 improved maize growth and the soil bacterial community structure under sterilized soil conditions [10]. The indigenous P-solubilizing bacteria *Bacillus cereus* SP11, SE, and T21 increased P solubility in Andisols [11]. In addition, *Enterobacter bugandensis* C5-7 improved maize growth under saline conditions [12].

Bacteria capable of associating with non-leguminous plants, such as maize, have attracted research interest in recent years [13,14,15]. The use of bacteria increases the population of beneficial microorganisms in soil, which is an important factor for increasing the content of soluble nutrients in soil [16,17,18]. Owing to their special metabolic capacity, purple nonsulfur bacteria (PNSB) adapt to various conditions [19]. In addition, PNSB can fix nitrogen [20], possess a high capacity to solubilize various phosphate fractions [21,22,23], solubilize different potassium-bearing minerals in soil [14,15,24], and secrete plant growth-promoting substances [25]. Moreover, PNSB have been applied to many crops and have shown promising effectiveness, such as potato and cucumber [26,27,28], pineapple [29], sesame [30], and canary melon [31]. In particular, among the PNSB, the four *R. palustris* strains VNW64, VNS89, TLS06, and VNW02 were selected because they had been previously characterized as phosphate-solubilizing PNSB with potential plant growth-promoting functions [32]. Moreover, a four-strain mixture was used to broaden functional stability under soil conditions and reduce the risk that the response would depend on a single strain [33]. Therefore, this study was conducted to determine the effects of phosphate-solubilizing purple nonsulfur bacteria on phosphorus nutrient dynamics, growth, and yield of hybrid maize grown in in-dyke alluvial soil collected from Chau Phu commune, An Giang province.

## 2. Results

### 2.1. Effects of Phosphorus-Solubilizing Rhodopseudomonas palustris on Soil Properties of Hybrid Maize Grown in In-Dyke Alluvial Soil

The pH_H2O_ values in treatments supplemented with PNSB combined with different fertilizer rates were all higher than those in the corresponding treatments without PNSB supplementation. Specifically, water-extracted pH in the PNSB-supplemented group ranged from 6.07 to 6.16, compared with 5.84 to 5.88 in the group receiving only the N, P, and K fertilizer background. Similarly, in the treatment without chemical fertilizer (0% N + 0% P + 0% K), PNSB supplementation also increased pH_H2O_ to 6.08 compared with 5.89 in the treatment without PNSB supplementation. However, pH_KCl_ values varied among treatments without a clear pattern, with the 0% N + 0% P + 0% K + PNSB treatment reaching the highest value of 5.06 (Table 1).

The presence of PNSB played an important role in increasing plant-available nitrogen (N) and soluble P content in soil. Specifically, NH_4_^+^ content increased markedly in all PNSB-supplemented treatments, reaching 16.3–18.6 mg kg^−1^ compared with 10.6–11.7 mg kg^−1^ in the treatments without PNSB supplementation. The 100% N + 100% P + 100% K + PNSB treatment had the highest NH_4_^+^ content (18.6 mg kg^−1^). In particular, soluble P content increased in treatments receiving chemical fertilizer at different rates combined with PNSB, reaching the highest value of 75.6 mg kg^−1^ in the 100% N + 100% P + 100% K + PNSB treatment. When the P fertilizer rate was reduced (100% N + 75% P + 100% K + PNSB, 100% N + 50% P + 100% K + PNSB, and 100% N + 25% P + 100% K + PNSB), soluble P content still reached 58.2–66.6 mg kg^−1^, higher than the values of 42.1–49.8 mg kg^−1^ in the treatments with the same chemical fertilizer rates but without PNSB supplementation (Table 1).

In contrast, insoluble P fractions, including Al-P, Ca-P, and Fe-P, all decreased in the treatments supplemented with PNSB. Ca-P and Fe-P contents were highest in the group of treatments receiving 100% N + 100% K combined with different P rates without PNSB supplementation (ranging from 151.4 to 168.1 mg kg^−1^ and 134.5 to 162.6 mg kg^−1^, respectively), but decreased in the PNSB-supplemented treatments (115.9–123.1 mg kg^−1^ and 112.4–125.9 mg kg^−1^, respectively). Similarly, Al-P content also decreased markedly in the PNSB-supplemented treatments, from 54.9–62.5 mg kg^−1^ to 37.3–56.9 mg kg^−1^ at the corresponding fertilizer rates (Table 1).

For EC, CEC, total P, total N, and OM, PNSB treatment and fertilizer rates during the experimental period did not lead to statistically significant differences among treatments (Table 1).

### 2.2. Effects of Phosphorus-Solubilizing Rhodopseudomonas palustris on Phosphorus Uptake of Hybrid Maize Grown in In-Dyke Alluvial Soil

Biomass of hybrid maize plant parts was highest in the 100% N + 100% P + 100% K + PNSB treatment, with 204.0 g pot^−1^ in stems and 117.9 g pot^−1^ in grains. Notably, when P was reduced by 25%, the 100% N + 75% P + 100% K + PNSB treatment still had higher stem and grain biomass than the control treatment receiving 100% N + 100% P + 100% K, with 144.6 compared with 139.5 g pot^−1^ in stems and 108.4 compared with 103.4 g pot^−1^ in grains. In addition, in soil without chemical fertilizer, PNSB supplementation clearly improved plant biomass. Typically, the 0% N + 0% P + 0% K + PNSB treatment produced stem and grain biomass of 94.8 and 85.2 g pot^−1^, respectively, exceeding the 56.5 and 73.1 g pot^−1^ recorded in the 0% N + 0% P + 0% K treatment. Moreover, root, leaf, husk, and cob biomass showed a similar trend (Table 2).

PNSB tended to increase P accumulation in hybrid maize plant parts. Specifically, under the chemical fertilizer background, the 100% N + 100% P + 100% K + PNSB treatment had higher P contents in roots, stems, leaves, grains, husks, and cobs than the 100% N + 100% P + 100% K treatment, with 0.354 compared with 0.313%, 0.420 compared with 0.353%, 0.222 compared with 0.186%, 0.394 compared with 0.358%, 0.219 compared with 0.144%, and 0.265 compared with 0.221%, respectively. In addition, the P-reduced treatment, 100% N + 75% P + 100% K + PNSB, still had grain P content of 0.401%, higher than that under 100% N + 100% P + 100% K. Under unfertilized conditions, the effect of PNSB was clearly reflected in the 0% N + 0% P + 0% K + PNSB treatment, where P contents in stems and grains reached 0.416 and 0.429%, respectively, exceeding 0.313 and 0.348% in the 0% N + 0% P + 0% K treatment. In particular, grain P content in the 0% N + 0% P + 0% K + PNSB treatment was also the highest value among all treatments (Table 3).

The 100% N + 100% P + 100% K + PNSB treatment achieved the highest P uptake in plant parts, specifically roots (0.024 g pot^−1^), stems (0.857 g pot^−1^), leaves (0.096 g pot^−1^), grains (0.465 g pot^−1^), husks (0.068 g pot^−1^), and cobs (0.093 g pot^−1^). Furthermore, the 100% N + 75% P + 100% K + PNSB treatment had greater P uptake in stems and grains than the 100% N + 100% P + 100% K treatment, with 0.556 compared with 0.492 g pot^−1^ in stems and 0.434 compared with 0.369 g pot^−1^ in grains. In addition, under unfertilized soil conditions, the 0% N + 0% P + 0% K + PNSB treatment had grain P uptake of 0.366 g pot^−1^, higher than the 0.254 g pot^−1^ recorded in the 0% N + 0% P + 0% K treatment. P uptake in other hybrid maize plant parts followed the same trend (Table 4).

**Figure 1:**
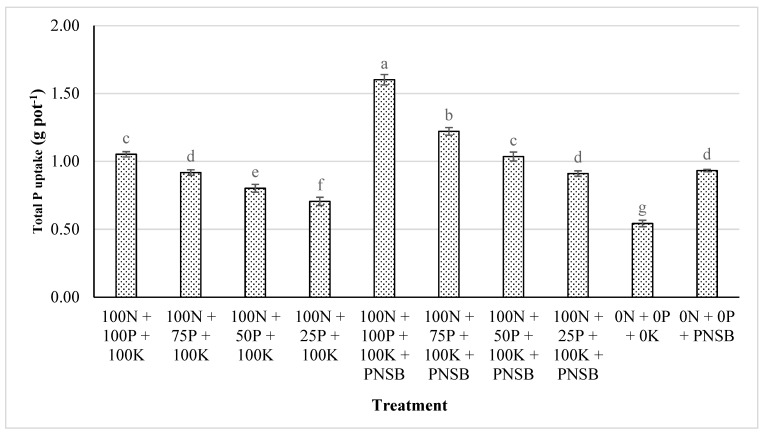
Effects of the four-strain mixture of phosphate-solubilizing purple nonsulfur bacteria *Rhodopseudomonas palustris* on total P uptake of hybrid maize grown in in-dyke alluvial soil. Note: Among columns, the same letters indicate no statistically significant difference; different letters indicate a statistically significant difference at the 5% level. PNSB: purple nonsulfur bacteria.

Total P uptake in the 100% N + 100% P + 100% K + PNSB and 100% N + 75% P + 100% K + PNSB treatments reached 1.60 and 1.22 g pot^−1^, respectively, higher than that in the control treatment of 100% N + 100% P + 100% K (1.05 g pot^−1^). All PNSB-supplemented treatments had higher values than the treatments receiving only the same chemical fertilizer rates. In addition, under unfertilized conditions, the 0% N + 0% P + 0% K + PNSB treatment reached 0.93 g pot^−1^, higher than the 0% N + 0% P + 0% K treatment, with 0.54 g pot^−1^ (Figure 1).

### 2.3. Effects of Phosphorus-Solubilizing Rhodopseudomonas palustris on Growth of Hybrid Maize Grown in In-Dyke Alluvial Soil

The absence of P fertilization reduced the growth characteristics of hybrid maize, including plant height, ear height, stem diameter, number of leaves, leaf length, and leaf width, compared with recommended P fertilization, with 184.9 cm, 104.1 cm, 1.92 cm, 16.0 leaves, 58.5 cm, and 6.35 cm at 100% P (100% N + 100% P + 100% K treatment) and 141.1 cm, 64.0 cm, 1.03 cm, 14.0 leaves, 55.5 cm, and 5.20 cm at 0% P (0% N + 0% P + 0% K treatment). The reduction was also reflected by the gradually decreasing P rates in the absence of PNSB supplementation (100% N + 75% P + 100% K, 100% N + 50% P + 100% K, and 100% N + 25% P + 100% K treatments). For the PNSB-supplemented treatments, growth characteristics were markedly improved. Specifically, the treatment receiving full chemical fertilizer combined with PNSB (100% N + 100% P + 100% K + PNSB) had the highest values for all traits, including plant height (207.6 cm), ear height (117.9 cm), stem diameter (2.17 cm), number of leaves (16.8 leaves), leaf length (62.4 cm), and leaf width (6.90 cm) (Table 5).

Notably, 100% N + 75% P + 100% K combined with PNSB resulted in plant height (188.5 cm), ear height (101.8 cm), stem diameter (1.81 cm), number of leaves (16.0 leaves), leaf length (58.9 cm), and leaf width (6.25 cm) that were equivalent to or higher than those under 100% N + 100% P + 100% K, with 184.9 cm, 104.1 cm, 1.92 cm, 16.0 leaves, 58.5 cm, and 6.35 cm, respectively. The potential of PNSB to substitute chemical P continued to be confirmed at lower P rates. Typically, the 100% N + 50% P + 100% K + PNSB treatment recorded higher plant height (182.9 cm), stem diameter (1.70 cm), and leaf width (5.78 cm) than the treatment with the same P rate but without PNSB supplementation, 100% N + 50% P + 100% K (176.7 cm, 1.61 cm, and 5.25 cm, respectively). Similarly, when only 25% P was applied, the 100% N + 25% P + 100% K + PNSB treatment had higher plant height, ear height, and number of leaves than the 100% N + 25% P + 100% K treatment, with 176.7 compared with 171.1 cm, 90.8 compared with 89.3 cm, and 16.0 compared with 15.3 leaves. For the unfertilized treatment, PNSB supplementation resulted in greater plant height, ear height, stem diameter, and number of leaves than without PNSB supplementation, with 160.2 compared with 141.1 cm, 70.8 compared with 64.0 cm, 1.15 compared with 1.03 cm, and 15.5 compared with 14.0 leaves, respectively (Table 5).

### 2.4. Effects of Phosphorus-Solubilizing Rhodopseudomonas palustris on Yield Components of Hybrid Maize Grown in In-Dyke Alluvial Soil

PNSB supplementation improved the SPAD index of hybrid maize leaves. Typically, the 100% N + 100% P + 100% K + PNSB and 100% N + 75% P + 100% K + PNSB treatments had the highest values (59.4–61.6), exceeding that of the 100% N + 100% P + 100% K treatment (53.9) (Table 6).

Reducing P fertilizer decreased yield components, including ear length, ear diameter, and number of kernels per row, but did not affect the number of rows per ear. Specifically, in treatments with P application compared with no P application, ear length was 16.6–18.0 cm compared with 12.9 cm, ear diameter was 2.61–3.26 compared with 2.16 cm, and the number of kernels per row was 25.8–37.8 compared with 24.0 kernels. Meanwhile, the number of rows per ear ranged from 8.50 to 12.5 rows among treatments (Table 6).

Applying 25% P combined with the PNSB mixture resulted in an ear diameter equivalent to that under 100% P, with 3.13 compared with 3.26 cm. However, ear length, number of kernels per row, and number of rows per ear did not differ significantly between the reduced-fertilizer treatments combined with the PNSB mixture (100% N + 100% P + 100% K + PNSB, 100% N + 75% P + 100% K + PNSB, 100% N + 50% P + 100% K + PNSB, and 100% N + 25% P + 100% K + PNSB) and the 100% N + 100% P + 100% K treatment. PNSB supplementation also played a positive role in the 0% N + 0% P + 0% K + PNSB treatment, significantly increasing ear length, ear diameter, and number of rows per ear compared with the 0% N + 0% P + 0% K treatment, with 14.5 compared with 12.9 cm, 3.15 compared with 2.16 cm, and 10.3 compared with 8.50 rows (Table 6).

### 2.5. Effects of Phosphorus-Solubilizing Rhodopseudomonas palustris on Grain Yield of Hybrid Maize Grown in In-Dyke Alluvial Soil

**Figure 2:**
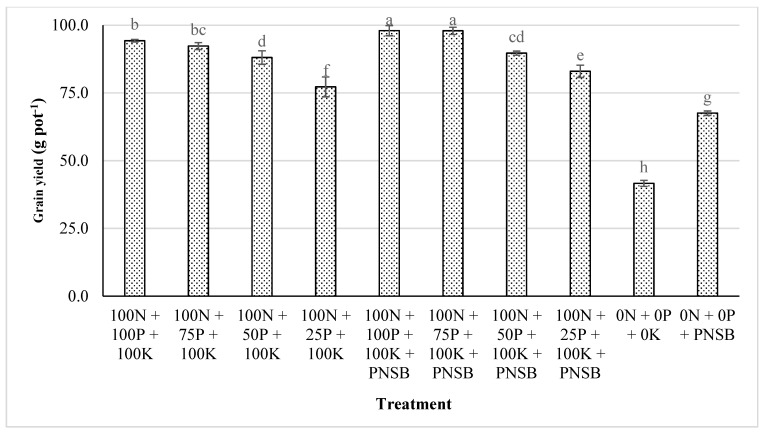
Effects of the four-strain mixture of phosphate-solubilizing purple nonsulfur bacteria *Rhodopseudomonas palustris* on yield of hybrid maize grown in in-dyke alluvial soil. Note: Among columns, the same letters indicate no statistically significant difference; different letters indicate a statistically significant difference at the 5% level. PNSB: purple nonsulfur bacteria.

Applying 100% N + 100% P + 100% K and 100% N + 75% P + 100% K according to the recommendation combined with supplementation of *R. palustris* strains VNW64, VNS89, TLS06, and VNW02 produced the highest maize yields. The treatment receiving 100% N + 100% P + 100% K according to the recommendation had a yield of 94.3 g pot^−1^, higher than the treatments receiving 50, 25, and 0% P, with 88.1, 77.3, and 41.7 g pot^−1^, respectively. The 100% N + 75% P + 100% K + PNSB treatment had a yield of 98.0 g pot^−1^, higher than the treatment receiving only 100% N + 75% P + 100% K (92.3 g pot^−1^). Moreover, the 100% N + 75% P + 100% K + PNSB treatment contributed to a higher yield than the treatment receiving only 100% N + 100% P + 100% K. The 100% N + 50% P + 100% K + PNSB treatment achieved a yield of 89.7 g pot^−1^, equivalent to the 100% N + 75% P + 100% K treatment. The 100% N + 25% P + 100% K + PNSB treatment achieved a hybrid maize yield of 83.0 g pot^−1^, higher than that of the 100% N + 25% P + 100% K treatment (77.3 g pot^−1^) (Figure 2).

The Pearson correlation heatmap (n = 40) showed that total P uptake was positively correlated with soluble P (*r* = 0.73, *p* < 0.001) and grain yield (*r* = 0.72, *p* < 0.001). Soluble P was negatively correlated with insoluble P fractions, especially Ca-P (*r* = −0.69, *p* < 0.001), followed by Al-P (*r* = −0.38, *p* = 0.015) and Fe-P (*r* = −0.37, *p* = 0.018) (Figure S1).

## 3. Discussion

PNSB supplementation was shown to improve soil pH (Table 1), which is consistent with previous studies showing that PNSB increased pH under in vitro, greenhouse, and field conditions in acid sulfate and saline soils [18,22,32]. In this study, PNSB contributed to improving the pH of in-dyke alluvial soil, providing further evidence of the diverse living conditions of PNSB [34]. Increased soil pH contributes to improved nutrient solubility in soil [35]. Specifically, PNSB-supplemented treatments increased soluble nutrient contents, such as plant-available N, soluble P, and exchangeable K (Table 1).

Among the three essential macronutrients NPK required for crops, N has the greatest effect on yield, whereas P and K have lower effects on yield [36]. In particular, P fertilizer-use efficiency in cereal crops is low, below 16% [37], causing nutrient imbalance. Therefore, residual P fertilizer is high and needs to be exploited to avoid wasting resources. The PNSB strains solubilized insoluble P into forms usable by crops, meaning that soluble P content in PNSB-supplemented treatments was higher than in treatments without PNSB supplementation, while Al-P, Fe-P, and Ca-P contents showed the opposite trend (Table 1). PNSB have been shown to decompose insoluble phosphate compounds in soil [17,22]. The classical explanation for phosphate solubilization by phosphate-solubilizing bacteria is the secretion of organic acids, which can acidify the immediate rhizosphere and dissolve insoluble phosphate compounds [38]. However, bulk soil pH measured after crop growth did not decrease in the current study (Table 1). This may reflect the net effect of multiple biological and chemical processes. Organic acid anions can mobilize P through ligand exchange and chelation of Al, Fe, and Ca without necessarily decreasing the final bulk soil pH [39,40]. In addition, PNSB metabolism, N transformation, root uptake balance, and microbial interactions may alter proton consumption and release, resulting in a higher measured bulk pH. Furthermore, the ability of PNSB to produce organic acids, siderophores, and other metabolites may promote the release of orthophosphate through chelation, ligand exchange, and dissolution of mineral-associated P [41]. Therefore, the decrease in mineral P fractions and the increase in soluble P in this study likely resulted from combined microbial and rhizosphere processes.

PNSB can adapt to diverse conditions [34], contributing to increased P nutrient uptake compared with treatments receiving only inorganic fertilizer without PNSB supplementation (Table 4). In addition, improved N and P availability can jointly enhance photosynthetic capacity, root growth, and biomass accumulation [42]. This is supported by the higher SPAD index, improved growth traits, greater dry biomass, and increased P uptake in inoculated treatments. The 100% N + 75% P + 100% K + PNSB treatment produced higher P uptake and grain yield than the full NPK treatment without PNSB, indicating that improved P mobilization and uptake efficiency contributed to maintaining maize productivity despite a 25% reduction in chemical P input. Previous studies have also shown that PNSB supplementation contributed to improved yields of upland crops [16,29]. Treatments supplemented with P-solubilizing PNSB, *R. palustris* VNW64, VNS89, TLS06, and VNW02, achieved higher yields than treatments receiving the same P rates but without PNSB supplementation (Figure 2). This is because bacterial supplementation releases substances during metabolism that enhance nutrient uptake, thereby increasing crop yield [43]. According to Majeed et al. [44], P-solubilizing bacterial strains also solubilized P in soil, contributing to increased pearl millet yield. In addition, supplementation with *Kluyvera cryocrescens* JBP1 reduced the recommended P fertilizer rate by 50% without reducing yield [45]. PNSB supplementation contributed to improved hybrid maize growth (Table 5). According to Chaouki et al. [46], P-solubilizing bacterial strains increased maize growth. Bacteria can stimulate plant growth through different mechanisms, such as producing plant hormones including salicylic acid, gibberellins, cytokinins, and indole-3-acetic acid [43], and increasing soluble nutrients for crops [46]. Supplementation with P-solubilizing bacterial strains increased ear length, ear diameter, and number of kernels per row of maize ears compared with the control treatment (Table 6).

Moreover, total P uptake was highest in the treatment receiving the recommended P rate combined with PNSB (Figure 1), indicating that total P uptake still has the potential to increase. Previous studies have also demonstrated increased P uptake in many crops, such as rice, canary melon, lemon balm, and pineapple [17,29,31,47]. In addition, P-solubilizing bacterial supplementation increased nutrient uptake in cultivation on soils with low soluble P content [46]. Noticeably, although the 0% N + 0% P + 0% K + PNSB treatment had the highest grain P concentration, its biomass and total P uptake were lower than those of fertilized PNSB treatments. Under severe nutrient limitation, plant growth is restricted, and nutrient concentration in tissues may appear high because of reduced biomass [48]. Therefore, P concentration alone does not fully describe plant nutritional performance; P uptake and biomass accumulation provide a more complete interpretation.

Ultimately, the four-strain PNSB mixture has potential agronomic and environmental value. Reducing chemical P fertilizer by 25% while maintaining or increasing grain yield could lower fertilizer costs and reduce the accumulation of residual P in soil. This may also help minimize the risk of P loss to surrounding water bodies. However, this study was conducted as a pot experiment using 12 kg of soil per pot and one maize plant per pot. Therefore, the results cannot be directly extrapolated to field conditions. In field environments, the performance of introduced PNSB may be affected by competition with native soil microorganisms, spatial heterogeneity, rainfall, irrigation, temperature fluctuation, and variation in root development. In addition, this study did not quantify the survival, colonization, or rhizosphere persistence of the introduced strains, and it did not compare sterilized and non-sterilized soil conditions. Therefore, the relative contribution of bacterial activity, bacterial biomass, and interactions with native microorganisms could not be fully separated. Therefore, practical application will require field validation, assessment of inoculant survival and colonization, evaluation of shelf life and formulation stability, and cost–benefit analysis under farmer-managed conditions. Future studies should also compare the four strains individually and in different combinations to determine whether the observed response is due to additive, synergistic, or functionally redundant effects among strains.

## 4. Materials and Methods

### 4.1. Materials

The purple nonsulfur bacterial strains *Rhodopseudomonas palustris* VNW64 (KY624606), VNS89 (KY624607), TLS06 (KY624609), and VNW02 (KY624605) possessing P-solubilizing function [32] were stored at −80 °C at the Faculty of Crop Science, College of Agriculture, Can Tho University. The phosphate-solubilizing capacities of VNW64, VNS89, TLS06, and VNW02 were shown as 2.82–3.30, 2.81–3.39, 2.67–3.69 and 2.57–3.50 PSI, respectively, under microaerobic light conditions. The strains were maintained as pure cultures and used as an equal-proportion mixture in this experiment.

In-dyke alluvial soil was collected from Chau Phu commune, An Giang province.

Hybrid maize variety CP 888: used in the experiment and supplied by C.P. SEEDS (Hanoi, Vietnam).

The experimental pots were black plastic pots with an upper diameter of 24 cm, a lower diameter of 23 cm, and a height of 24 cm.

The fertilizers used were urea (46% N, Phu My Company, Ho Chi Minh City, Vietnam), superphosphate (16% P_2_O_5_, Long Thanh Company, Ho Chi Minh City, Vietnam), and potassium fertilizer (60% K_2_O, Phu My Company, Ho Chi Minh City, Vietnam).

### 4.2. Methods

Experimental design: The experiment was arranged in a randomized complete block design with 10 treatments and four replicates per treatment, with each replicate corresponding to one pot, including: (i) 100% N + 100% P + 100% K, (ii) 100% N + 75% P + 100% K, (iii) 100% N + 50% P + 100% K, (iv) 100% N + 25% P + 100% K, (v) 100% N + 100% P + 100% K + a four-strain mixture of P-solubilizing purple nonsulfur bacteria (PNSB), (vi) 100% N + 75% P + 100% K + PNSB, (vii) 100% N + 50% P + 100% K + PNSB, (viii) 100% N + 25% P + 100% K + PNSB, (ix) 0% N + 0% P + 0% K, and (x) 0% N + 0% P + 0% K + PNSB. The pot experiment was conducted under net house conditions at the Faculty of Crop Science, College of Agriculture, Can Tho University, from February to June. During the experimental period, the average temperature was 37.3 °C, relative humidity was 61.8%, and plants were grown under natural photoperiod conditions of approximately 13 h light day^−1^. Pots were arranged to minimize shading effects and were rotated periodically within each block to reduce positional variation. The C11 pot (Thanh Cong Company, Ho Chi Minh City, Vietnam) was 28 × 23 × 22 cm for top, bottom, and height, respectively.

Seed treatment: Maize seeds were sterilized with 70% ethanol for 3 min and 1% sodium hypochlorite solution for 10 min. The maize seeds were then rinsed three times with sterilized demineralized water before being incubated in the dark for 24 h for germination. Germinated hybrid maize seeds were placed in glass beakers containing a prepared mixed solution of P-solubilizing purple nonsulfur bacteria, *Rhodopseudomonas palustris* VNW64, VNS89, TLS06, and VNW02, at a density of 10^8^ CFU/mL, and the germinated hybrid maize seeds were incubated for 1 h. For treatments without bacterial inoculation, maize seeds were placed in glass beakers containing sterilized distilled water. The beakers were shaken at 60 rpm for 1 h prior to the seeds being dried in a microbiological culture cabinet for 1 h before sowing. The seeds were then planted in experimental pots, with two seeds per pot, and thinned to one plant per pot 10–15 days later. Therefore, for each seed in the pot, the bacterial density reached 0.083 × 10^5^ CFU g^−1^ dry soil. PNSB was applied at 15 mL on 24, 32, and 61 days after sowing (DAS), and the density reached 3.75 × 10^5^ CFU g^−1^ dry soil. The total PNSB was 3.833 × 10^5^ CFU g^−1^ dry soil.

*Soil:* In-dyke alluvial soil was collected from the 0–20 cm layer, air-dried, cleared of plant residues, and accurately weighed at 12 kg for each pot.

The inorganic fertilizer formula applied to hybrid maize was 200N-90P_2_O_5_-80K_2_O.

Growth parameters: Growth parameters were measured at 108 DAS and are described in detail below.

*Plant height (cm):* Measured from the base to the growing tip of the plant.

*Ear height (cm):* Measured from the soil surface to the position of the productive ear.

*Stem diameter (cm):* Measured at the base, middle, and top of the stem to calculate the average value.

*Number of leaves per plant:* All leaves on the plant were counted.

*Leaf length (cm):* Measured from the leaf base to the leaf tip of the fifth leaf counted from the top.

*Leaf width (cm):* Measured at the middle of the fifth leaf counted from the top.

Hybrid maize yield (g/plant): The grain weight of each pot was weighed at 108 DAS.

*Fresh and dry biomass (g/plant):* The entire fresh weight of the plant was weighed by plant part (root, stem, leaf, grain, husk, and cob) to determine fresh weight. Subsequently, all fresh biomass was dried at 70 °C for 72 h to determine dry biomass.

Yield components: Yield components were determined at 108 DAS, with four plants measured for each treatment from four replicates.

*Ear length (cm):* The length of each ear was measured from the first to the last grain-bearing point.

*Ear diameter (cm):* The diameter of each ear was measured at three positions to calculate the average value.

*Number of rows per ear (rows):* All rows with complete grains on the ear were counted.

*Number of kernels per row (kernels):* All kernels on one row of each ear were counted, and all rows were counted.

According to Sparks et al. [49], soil sample analyses were conducted as follows: pH_H2O_ and pH_KCl_ were extracted with distilled water (pH_KCl_ extracted with KCl) at a ratio of 1:2.5, centrifuged at 2000 rpm for 10 min, filtered through filter paper to obtain the clear solution, and measured using a pH meter; after measuring pH_H2O_, the solution was used to measure EC using an EC meter. For total P content, soil was digested with a concentrated H_2_SO_4_-HClO_4_ mixture; soluble P was extracted with 1.0 M NH_4_F and 0.5 M HCl using the Bray 2 method; and iron-bound P (Fe-P), aluminum-bound P (Al-P), and calcium-bound P (Ca-P) were extracted with 0.1 M NaOH, 0.5 M NH_4_F, and 0.25 M H_2_SO_4_, respectively. All of the P fractions were determined colorimetrically at 880 nm. NH_4_^+^ content was measured by extracting soil with 2.0 M KCl and colorimetrically determined using a spectrophotometer at 650 nm (AAS-AA7000, Shimadzu, Kyoto, Japan). Total N content was determined by the Kjeldahl distillation method from a digestion solution prepared with a concentrated H_2_SO_4_:CuSO_4_:Se mixture at a ratio of 100:10:1. Organic matter was measured using the Walkley-Black method, with oxidation by concentrated H_2_SO_4_-K_2_Cr_2_O_7_ before titration with FeSO_4_. Cation exchange capacity (CEC) was extracted with 0.1 M BaCl_2_ and titrated with 0.01 M EDTA.

P content in roots, stems, leaves, grains, husks, and cobs was analyzed as follows: samples were digested with 6 g salicylic acid, 18 mL water, and 100 mL concentrated H_2_SO_4_. H_2_O_2_ was used for oxidation by adding 1 mL every 30 min after cooling. P content (%) was determined colorimetrically using a spectrophotometer at 880 nm [50]. P uptake in roots (g plant^−1^) = P content in roots (%) × root dry biomass (g plant^−1^). P uptake in stems, leaves, grains, husks, and cobs was calculated similarly.

### 4.3. Data Analysis

Data were compiled and processed using Microsoft Excel 2013. Statistical analysis was performed using SPSS version 16.0, and Duncan’s test was used to compare treatment means at the 5% significance level.

## 5. Conclusions

Supplementation with purple nonsulfur bacteria capable of solubilizing P, *Rhodopseudomonas palustris* VNW64, VNS89, TLS06, and VNW02, increased soluble P content, reduced the contents of insoluble P forms including Al-P, Fe-P, and Ca-P, and positively affected P nutrient uptake by hybrid maize. The four-strain mixture of P-solubilizing purple nonsulfur bacteria helped reduce chemical P fertilizer applied to maize by 25% while maintaining higher growth and yield than the treatment receiving 100% of the recommended P rate. These findings suggest that the selected PNSB mixture has potential to reduce chemical P fertilizer input by 25% under the conditions of this pot experiment. However, field validation is required to confirm inoculant performance, persistence, economic feasibility, and fertilizer-saving potential under practical cultivation conditions.

## Figures and Tables

**Table 1:** Effects of the four-strain mixture of phosphate-solubilizing purple nonsulfur bacteria *R. palustris* on properties of in-dyke alluvial soil cultivated with hybrid maize.

Treatment	pH_H2O_	pH_KCl_	EC (mS cm^−1^)	CEC (meq 100 g^−1^)	NH_4_^+^ (mg kg^−1^)	OM (%C)
100 N + 100 P + 100 K	5.88 ± 0.05 ^b^	4.97 ± 0.03 ^a–d^	0.24 ± 0.05 ^a^	18.1 ± 0.6 ^a^	11.1 ± 0.3 ^c^	2.12 ± 0.09 ^ab^
100 N + 75 P + 100 K	5.85 ± 0.04 ^b^	4.87 ± 0.11 ^cd^	0.24 ± 0.03 ^a^	16.2 ± 1.6 ^a^	10.6 ± 0.6 ^c^	1.92 ± 0.22 ^ab^
100 N + 50 P + 100 K	5.84 ± 0.07 ^b^	4.94 ± 0.12 ^a–d^	0.25 ± 0.02 ^a^	17.2 ± 1.5 ^a^	11.7 ± 1.1 ^c^	2.32 ± 0.35 ^a^
100 N + 25 P + 100 K	5.85 ± 0.10 ^b^	4.90 ± 0.06 ^bcd^	0.26 ± 0.02 ^a^	15.4 ± 0.9 ^a^	11.3 ± 1.0 ^c^	2.00 ± 0.24 ^ab^
100 N + 100 P + 100 K + PNSB	6.16 ± 0.25 ^a^	5.02 ± 0.03 ^abc^	0.28 ± 0.11 ^a^	16.5 ± 2.2 ^a^	18.6 ± 1.2 ^a^	2.06 ± 0.30 ^ab^
100 N + 75 P + 100 K + PNSB	6.09 ± 0.07 ^a^	4.84 ± 0.10 ^d^	0.25 ± 0.04 ^a^	17.0 ± 1.8 ^a^	16.3 ± 1.1 ^b^	1.78 ± 0.17 ^b^
100 N + 50 P + 100 K + PNSB	6.07 ± 0.03 ^a^	4.85 ± 0.08 ^d^	0.25 ± 0.07 ^a^	15.8 ± 0.8 ^a^	16.4 ± 0.7 ^b^	1.95 ± 0.20 ^ab^
100 N + 25 P + 100 K + PNSB	6.12 ± 0.05 ^a^	4.93 ± 0.16 ^a–d^	0.24 ± 0.09 ^a^	16.1 ± 2.2 ^a^	16.4 ± 0.6 ^b^	2.27 ± 0.42 ^a^
0 N + 0 P + 0 K	5.89 ± 0.05 ^b^	5.04 ± 0.02 ^ab^	0.19 ± 0.02 ^a^	17.4 ± 0.8 ^a^	11.4 ± 0.6 ^c^	2.04 ± 0.24 ^ab^
0 N + 0 P + PNSB	6.08 ± 0.03 ^a^	5.06 ± 0.06 ^a^	0.23 ± 0.02 ^a^	17.3 ± 1.0 ^a^	16.1 ± 0.4 ^b^	2.17 ± 0.23 ^ab^
Significance level	*	*	ns	ns	*	ns
CV (%)	1.64	1.89	22.8	8.63	5.96	12.7
**Treatment**	**P_total_ (%)**	**N_total_** **(%)**	**P_soluble_** **(mg P kg^−1^)**	**Al-P** **(mg P kg^−1^)**	**Ca-P** **(mg P kg^−1^)**	**Fe-P** **(mg P kg^−1^)**
100 N + 100 P + 100 K	0.052 ± 0.016 ^a^	0.208 ± 0.029 ^a^	49.8 ± 3.3 ^e^	58.0 ± 1.5 ^bc^	168.1 ± 2.4 ^a^	162.6 ± 5.0 ^a^
100 N + 75 P + 100 K	0.046 ± 0.007 ^a^	0.215 ± 0.016 ^a^	49.7 ± 12.9 ^e^	60.2 ± 5.0 ^ab^	151.4 ± 14.9 ^ab^	137.3 ± 4.5 ^b^
100 N + 50 P + 100 K	0.048 ± 0.017 ^a^	0.195 ± 0.042 ^a^	48.9 ± 0.8 ^e^	62.5 ± 5.9 ^a^	152.7 ± 19.1 ^ab^	134.5 ± 5.6 ^b^
100 N + 25 P + 100 K	0.065 ± 0.011 ^a^	0.185 ± 0.022 ^a^	42.1 ± 3.4 ^f^	54.9 ± 2.4 ^cd^	152.3 ± 7.9 ^ab^	138.4 ± 6.1 ^b^
100 N + 100 P + 100 K + PNSB	0.059 ± 0.013 ^a^	0.238 ± 0.017 ^a^	75.6 ± 3.5 ^a^	56.9 ± 0.5 ^bcd^	123.1 ± 2.6 ^c^	125.9 ± 8.5 ^c^
100 N + 75 P + 100 K + PNSB	0.069 ± 0.007 ^a^	0.222 ± 0.021 ^a^	66.6 ± 2.4 ^b^	50.1 ± 0.7 ^e^	115.9 ± 14.6 ^c^	112.4 ± 3.9 ^d^
100 N + 50 P + 100 K + PNSB	0.064 ± 0.008 ^a^	0.225 ± 0.009 ^a^	62.9 ± 2.5 ^bc^	42.7 ± 0.5 ^f^	116.0 ± 19.9 ^c^	119.3 ± 8.0 ^cd^
100 N + 25 P + 100 K + PNSB	0.056 ± 0.013 ^a^	0.210 ± 0.027 ^a^	58.2 ± 0.7 ^cd^	37.3 ± 1.3 ^g^	122.7 ± 10.4 ^c^	121.9 ± 3.5 ^c^
0 N + 0 P + 0 K	0.056 ± 0.003 ^a^	0.197 ± 0.008 ^a^	53.2 ± 0.6 ^de^	53.5 ± 0.3 ^de^	141.2 ± 7.1 ^b^	88.1 ± 2.2 ^e^
0 N + 0 P + PNSB	0.057 ± 0.003 ^a^	0.205 ± 0.008 ^a^	62.6 ± 0.7 ^bc^	39.5 ± 1.0 ^fg^	121.4 ± 1.1 ^c^	82.1 ± 3.3 ^e^
Significance level	ns	ns	*	*	*	*
CV (%)	19.07	3.18	8.18	5.19	8.77	4.44

Note: Within the same column, values followed by the same letter are not significantly different; *: significant difference at 5%; ns: not significant. PNSB: purple nonsulfur bacteria.

**Table 2:** Effects of the four-strain mixture of phosphate-solubilizing purple nonsulfur bacteria *R. palustris* on biomass of hybrid maize grown in in-dyke alluvial soil.

Treatment	Biomass (g pot^−1^)					
	Root	Stem	Leaf	Grain	Husk	Cob
100 N + 100 P + 100 K	6.17 ± 0.07 ^b^	139.5 ± 2.5 ^b^	39.0 ± 0.6 ^c^	103.4 ± 2.9 ^c^	19.9 ± 1.0 ^c^	32.2 ± 1.0 ^b^
100 N + 75 P + 100 K	6.20 ± 0.05 ^b^	106.7 ± 5.8 ^d^	38.7 ± 1.4 ^c^	102.0 ± 4.1 ^cd^	19.2 ± 1.0 ^c^	31.7 ± 0.9 ^b^
100 N + 50 P + 100 K	5.43 ± 0.13 ^de^	82.5 ± 3.5 ^f^	37.7 ± 1.0 ^c^	90.1 ± 2.4 ^e^	19.1 ± 1.2 ^c^	31.3 ± 1.7 ^bc^
100 N + 25 P + 100 K	5.30 ± 0.05 ^e^	68.4 ± 3.4 ^g^	37.6 ± 0.9 ^c^	84.8 ± 0.8 ^f^	14.9 ± 0.6 ^e^	30.2 ± 0.4 ^c^
100 N + 100 P + 100 K + PNSB	6.74 ± 0.17 ^a^	204.0 ± 8.8 ^a^	43.2 ± 2.2 ^a^	117.9 ± 1.4 ^a^	31.3 ± 0.6 ^a^	34.8 ± 0.9 ^a^
100 N + 75 P + 100 K + PNSB	6.29 ± 0.18 ^b^	144.6 ± 2.5 ^b^	41.2 ± 0.7 ^b^	108.4 ± 1.4 ^b^	28.5 ± 1.7 ^b^	32.4 ± 0.9 ^b^
100 N + 50 P + 100 K + PNSB	5.85 ± 0.16 ^c^	114.6 ± 2.9 ^c^	38.7 ± 0.3 ^c^	98.9 ± 3.3 ^d^	20.4 ± 0.4 ^c^	31.8 ± 0.1 ^b^
100 N + 25 P + 100 K + PNSB	5.52 ± 0.04 ^d^	103.7 ± 2.4 ^d^	35.3 ± 0.9 ^d^	91.8 ± 3.5 ^e^	19.0 ± 2.0 ^c^	32.2 ± 1.2 ^b^
0 N + 0 P + 0 K	4.49 ± 0.04 ^g^	56.5 ± 2.5 ^h^	34.4 ± 0.4 ^d^	73.1 ± 2.0 ^g^	11.9 ± 0.6 ^f^	21.0 ± 0.8 ^e^
0 N + 0 P + PNSB	5.06 ± 0.14 ^f^	94.8 ± 1.7 ^e^	37.9 ± 0.7 ^c^	85.2 ± 1.2 ^f^	16.6 ± 0.3 ^d^	24.7 ± 0.3 ^d^
Significance level	*	*	*	*	*	*
CV (%)	2.05	3.70	2.77	2.66	5.34	3.05

Note: Within the same column, values followed by the same letter are not significantly different; *: significant difference at 5%. PNSB: purple nonsulfur bacteria.

**Table 3:** Effects of the four-strain mixture of phosphate-solubilizing purple nonsulfur bacteria *R. palustris* on P content of hybrid maize grown in in-dyke alluvial soil.

Treatment	P Content (%)					
	Root	Stem	Leaf	Grain	Husk	Cob
100 N + 100 P + 100 K	0.313 ± 0.009 ^cd^	0.353 ± 0.026 ^c^	0.186 ± 0.03 ^cd^	0.358 ± 0.021 ^c^	0.144 ± 0.008 ^d^	0.221 ± 0.014 ^bcd^
100 N + 75 P + 100 K	0.301 ± 0.005 ^cd^	0.357 ± 0.011 ^bc^	0.179 ± 0.05 ^d^	0.345 ± 0.022 ^c^	0.120 ± 0.004 ^e^	0.238 ± 0.007 ^b^
100 N + 50 P + 100 K	0.310 ± 0.021 ^cd^	0.361 ± 0.026 ^bc^	0.180 ± 0.06 ^d^	0.352 ± 0.018 ^c^	0.165 ± 0.003 ^bc^	0.229 ± 0.018 ^bc^
100 N + 25 P + 100 K	0.307 ± 0.010 ^cd^	0.346 ± 0.018 ^c^	0.172 ± 0.08 ^de^	0.357 ± 0.017 ^c^	0.104 ± 0.010 ^f^	0.231 ± 0.020 ^bc^
100 N + 100 P + 100 K + PNSB	0.354 ± 0.031 ^b^	0.420 ± 0.008 ^a^	0.222 ± 0.002 ^a^	0.394 ± 0.014 ^b^	0.219 ± 0.007 ^a^	0.265 ± 0.015 ^a^
100 N + 75 P + 100 K + PNSB	0.324 ± 0.015 ^c^	0.384 ± 0.025 ^b^	0.217 ± 0.007 ^ab^	0.401 ± 0.011 ^b^	0.171 ± 0.007 ^b^	0.227 ± 0.002 ^bcd^
100 N + 50 P + 100 K + PNSB	0.320 ± 0.005 ^c^	0.375 ± 0.013 ^bc^	0.215 ± 0.011 ^ab^	0.398 ± 0.025 ^b^	0.175 ± 0.007 ^b^	0.234 ± 0.011 ^bc^
100 N + 25 P + 100 K + PNSB	0.316 ± 0.004 ^c^	0.359 ± 0.025 ^bc^	0.218 ± 0.030 ^ab^	0.372 ± 0.017 ^bc^	0.170 ± 0.002 ^b^	0.216 ± 0.009 ^cd^
0 N + 0 P + 0 K	0.292 ± 0.015 ^d^	0.313 ± 0.008 ^d^	0.156 ± 0.007 ^e^	0.348 ± 0.026 ^c^	0.110 ± 0.006 ^ef^	0.153 ± 0.007 ^e^
0 N + 0 P + PNSB	0.377 ± 0.007 ^a^	0.416 ± 0.014 ^a^	0.202 ± 0.013 ^bc^	0.429 ± 0.003 ^a^	0.159 ± 0.009 ^c^	0.208 ± 0.012 ^d^
Significance level	*	*	*	*	*	*
CV (%)	4.58	5.09	6.12	4.95	4.60	5.57

Note: Within the same column, values followed by the same letter are not significantly different; *: significant difference at 5%. PNSB: purple nonsulfur bacteria.

**Table 4:** Effects of the four-strain mixture of phosphate-solubilizing purple nonsulfur bacteria *R. palustris* on P uptake of hybrid maize grown in in-dyke alluvial soil.

Treatment	Uptake (g pot^−1^)					
	Root	Stem	Leaf	Grain	Husk	Cob
100 N + 100 P + 100 K	0.020 ± 0.001 ^bc^	0.492 ± 0.034 ^c^	0.072 ± 0.001 ^de^	0.369 ± 0.016 ^cd^	0.029 ± 0.003 ^ef^	0.071 ± 0.003 ^b^
100 N + 75 P + 100 K	0.019 ± 0.001 ^cd^	0.381 ± 0.016 ^e^	0.070 ± 0.004 ^de^	0.351 ± 0.012 ^d^	0.023 ± 0.001 ^g^	0.075 ± 0.003 ^b^
100 N + 50 P + 100 K	0.017 ± 0.002 ^e^	0.298 ± 0.028 ^f^	0.068 ± 0.003 ^e^	0.316 ± 0.014 ^ef^	0.032 ± 0.002 ^de^	0.072 ± 0.009 ^b^
100 N + 25 P + 100 K	0.016 ± 0.001 ^e^	0.236 ± 0.017 ^g^	0.065 ± 0.004 ^e^	0.303 ± 0.015 ^f^	0.016 ± 0.001 ^h^	0.070 ± 0.007 ^b^
100 N + 100 P + 100 K + PNSB	0.024 ± 0.003 ^a^	0.857 ± 0.031 ^a^	0.096 ± 0.006 ^a^	0.465 ± 0.016 ^a^	0.068 ± 0.002 ^a^	0.093 ± 0.008 ^a^
100 N + 75 P + 100 K + PNSB	0.021 ± 0.002 ^b^	0.556 ± 0.032 ^b^	0.090 ± 0.004 ^b^	0.434 ± 0.029 ^b^	0.049 ± 0.004 ^b^	0.074 ± 0.002 ^b^
100 N + 50 P + 100 K + PNSB	0.019 ± 0.001 ^cd^	0.430 ± 0.011 ^d^	0.083 ± 0.004 ^bc^	0.394 ± 0.029 ^c^	0.036 ± 0.001 ^c^	0.074 ± 0.003 ^b^
100 N + 25 P + 100 K + PNSB	0.018 ± 0.001 ^de^	0.372 ± 0.019 ^e^	0.077 ± 0.009 ^cd^	0.342 ± 0.028 ^de^	0.033 ± 0.003 ^cd^	0.069 ± 0.005 ^b^
0 N + 0 P + 0 K	0.013 ± 0.001 ^f^	0.177 ± 0.010 ^h^	0.054 ± 0.002 ^f^	0.254 ± 0.021 ^g^	0.013 ± 0.001 ^h^	0.032 ± 0.002 ^d^
0 N + 0 P + PNSB	0.019 ± 0.001 ^bcd^	0.394 ± 0.006 ^e^	0.077 ± 0.005 ^cd^	0.366 ± 0.005 ^d^	0.026 ± 0.001 ^fg^	0.052 ± 0.003 ^c^
Significance level	*	*	*	*	*	*
CV (%)	6.21	5.38	6.18	5.19	6.57	7.39

Note: Within the same column, values followed by the same letter are not significantly different; *: significant difference at 5%. PNSB: purple nonsulfur bacteria.

**Table 5:** Effects of the four-strain mixture of phosphate-solubilizing purple nonsulfur bacteria *R. palustris* on growth of hybrid maize grown in in-dyke alluvial soil.

Treatment	Plant Height (cm)	Ear Height (cm)	Stem Diameter (cm)	Number of Leaves (leaves)	Leaf Length (cm)	Leaf Width (cm)
100 N + 100 P + 100 K	184.9 ± 4.1 ^c^	104.1 ± 3.6 ^b^	1.92 ± 0.03 ^b^	16.0 ± 0.0 ^b^	58.5 ± 0.4 ^b^	6.35 ± 0.47 ^b^
100 N + 75 P + 100 K	178.3 ± 2.0 ^d^	98.7 ± 1.9 ^c^	1.64 ± 0.02 ^de^	16.0 ± 0.0 ^b^	57.0 ± 0.9 ^bcd^	5.93 ± 0.34 ^bc^
100 N + 50 P + 100 K	176.7 ± 1.3 ^d^	93.2 ± 1.2 ^d^	1.61 ± 0.06 ^e^	15.8 ± 0.5 ^b^	56.1 ± 1.0 ^cd^	5.25 ± 0.29 ^d^
100 N + 25 P + 100 K	171.1 ± 4.1 ^e^	89.3 ± 1.2 ^e^	1.46 ± 0.05 ^f^	15.3 ± 0.5 ^b^	54.0 ± 1.3 ^e^	4.75 ± 0.19 ^e^
100 N + 100 P + 100 K + PNSB	207.6 ± 0.9 ^a^	117.9 ± 4.8 ^a^	2.17 ± 0.08 ^a^	16.8 ± 1.0 ^a^	62.4 ± 2.6 ^a^	6.90 ± 0.2 ^a^
100 N + 75 P + 100 K + PNSB	188.5 ± 1.9 ^a^	101.8 ± 1.0 ^bc^	1.81 ± 0.07 ^c^	16.0 ± 0.0 ^b^	58.9 ± 0.2 ^b^	6.25 ± 0.25 ^b^
100 N + 50 P + 100 K + PNSB	182.9 ± 2.7 ^c^	92.5 ± 0.9 ^de^	1.70 ± 0.05 ^d^	15.5 ± 0.6 ^b^	57.7 ± 0.5 ^bc^	5.78 ± 0.39 ^c^
100 N + 25 P + 100 K + PNSB	176.7 ± 1.2 ^d^	90.8 ± 1.2 ^de^	1.63 ± 0.07 ^de^	16.0 ± 0.0 ^b^	56.3 ± 1.6 ^cd^	5.55 ± 0.38 ^cd^
0 N + 0 P + 0 K	141.1 ± 1.6 ^g^	64.0 ± 0.4 ^g^	1.03 ± 0.03 ^h^	14.0 ± 0.0 ^c^	55.5 ± 0.9 ^de^	5.20 ± 0.00 ^d^
0 N + 0 P + PNSB	160.2 ± 0.3 ^f^	70.8 ± 0.9 ^f^	1.15 ± 0.02 ^g^	15.5 ± 0.6 ^b^	55.3 ± 0.3 ^de^	5.63 ± 0.05 ^cd^
Significance level	*	*	*	*	*	*
CV (%)	1.31	2.35	3.30	2.91	2.10	5.07

Note: Within the same column, values followed by the same letter are not significantly different; *: significant difference at 5%. PNSB: purple nonsulfur bacteria.

**Table 6:** Effects of the four-strain mixture of phosphate-solubilizing purple nonsulfur bacteria *R. palustris* on SPAD index and yield components of hybrid maize grown in in-dyke alluvial soil.

Treatment	SPAD	Ear Length (cm)	Ear Diameter (cm)	Number of Rows per Ear (Rows)	Number of Kernels per Row (Kernels)
100 N + 100 P + 100 K	53.9 ± 1.1 ^c^	18.0 ± 0.9 ^bcd^	3.26 ± 0.04 ^ab^	11.5 ± 1.0 ^ab^	37.5 ± 1.9 ^a^
100 N + 75 P + 100 K	52.1 ± 1.6 ^c^	17.8 ± 0.6 ^cd^	3.00 ± 0.17 ^cd^	12.5 ± 1.0 ^a^	37.8 ± 1.3 ^a^
100 N + 50 P + 100 K	51.8 ± 3.2 ^c^	17.6 ± 1.0 ^d^	2.88 ± 0.06 ^d^	11.5 ± 1.0 ^ab^	34.3 ± 4.9 ^b^
100 N + 25 P + 100 K	47.3 ± 0.4 ^d^	16.6 ± 0.8 ^e^	2.61 ± 0.21 ^e^	12.5 ± 1.0 ^a^	25.8 ± 0.5 ^cd^
100 N + 100 P + 100 K + PNSB	61.6 ± 1.7 ^a^	19.4 ± 0.1 ^a^	3.42 ± 0.20 ^a^	12.0 ± 1.6 ^ab^	39.8 ± 1.3 ^a^
100 N + 75 P + 100 K + PNSB	59.4 ± 0.9 ^a^	18.7 ± 0.2 ^abc^	3.44 ± 0.10 ^a^	12.0 ± 0.0 ^ab^	39.3 ± 0.5 ^a^
100 N + 50 P + 100 K + PNSB	56.8 ± 3.2 ^b^	18.9 ± 1.0 ^ab^	3.28 ± 0.06 ^ab^	11.5 ± 1.0 ^ab^	38.3 ± 1.3 ^a^
100 N + 25 P + 100 K + PNSB	56.5 ± 0.1 ^b^	18.2 ± 0.2 ^bcd^	3.13 ± 0.06 ^bc^	11.5 ± 1.9 ^ab^	36.5 ± 2.9 ^ab^
0 N + 0 P + 0 K	31.4 ± 0.6 ^f^	12.9 ± 0.4 ^g^	2.16 ± 0.10 ^f^	8.50 ± 0.6 ^c^	24.0 ± 0.0 ^d^
0 N + 0 P + PNSB	43.7 ± 0.3 ^e^	14.5 ± 0.2 ^f^	3.15 ± 0.02 ^bc^	10.3 ± 0.5 ^b^	28.0 ± 0.0 ^c^
Significance level	*	*	*	*	*
CV (%)	3.25	3.74	4.00	9.60	6.00

Note: Within the same column, values followed by the same letter are not significantly different; *: significant difference at 5%. PNSB: purple nonsulfur bacteria.

## Data Availability

The data presented in this study are available within the article. Additional data supporting the findings of this study are available from the corresponding author upon reasonable request.

## Supplementary Materials

The following supporting information can be downloaded at: https://www.mdpi.com/article/10.3390/plants15152312/s1.

## Abbreviations

The following abbreviations are used in this manuscript:

Al-P	aluminum-bound phosphorus
Ca-P	calcium-bound phosphorus
CEC	cation exchange capacity
CFU	colony-forming units
DAS	days after sowing
EC	electrical conductivity
Fe-P	iron-bound phosphorus
K	potassium
N	nitrogen
NH_4_^+^	ammonium
N_total_	total nitrogen
OM	organic matter
P	phosphorus
pH_H2O_	soil pH measured in water
pH_KCl_	soil pH measured in potassium chloride
PNSB	purple nonsulfur bacteria
P_total_	total phosphorus
SPAD	Soil–Plant Analysis Development chlorophyll index
