# Supplementary material for: Effects of Phosphorus-Solubilizing Rhodopseudomonas palustris VNW64, VNS89, TLS06, and VNW02 on Phosphorus Nutrient Dynamics and Yield of Hybrid Maize Grown in In-Dyke Alluvial Soil
Source: Plants (Basel). 2026 Jul 28;15(15):2312. doi: 10.3390/plants15152312 (PMC13468074; doi:10.3390/plants15152312)
Supplement: Supplementary file 1 [file plants-15-02312-s001.zip › plants-4389260-supplementary.pdf]

Supplementary Material

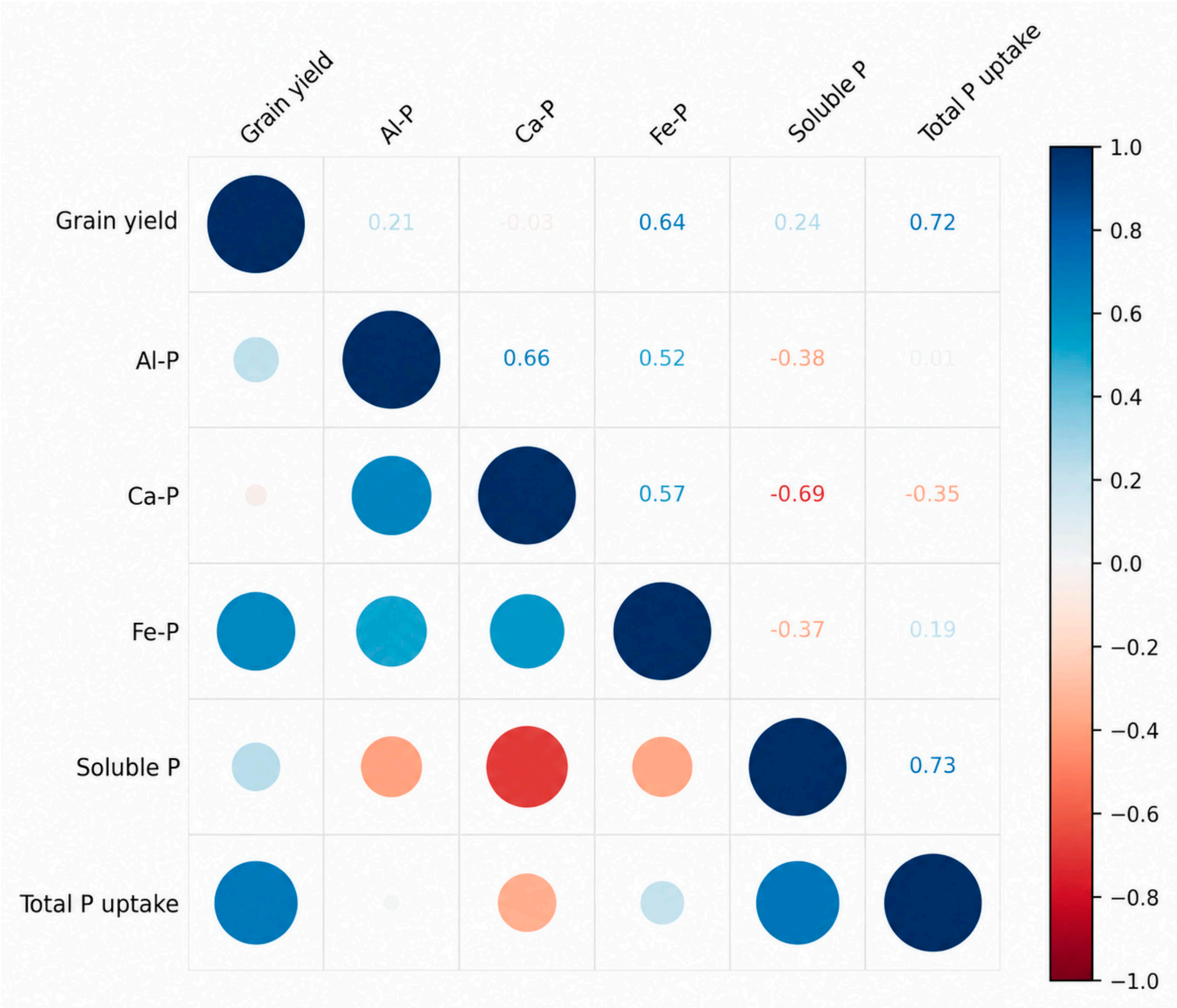

Figure S1. Pearson correlation heatmap among grain yield, soil P fractions, and total P uptake of hybrid maize grown in in-dyke alluvial soil. Blue and red circles indicate positive and negative correlations, respectively, while circle size and color intensity represent the strength of the Pearson correlation coefficient (*r*).
